# Percutaneous atrial septal defect closure in limited-resource setting: a decade-long experience from Ethiopia

**DOI:** 10.3389/fcvm.2025.1550693

**Published:** 2025-02-18

**Authors:** Mohammed Nasir, Kefelegn Dejene, Mohammed Bedru, Sura Markos

**Affiliations:** ^1^ Pediatrics and Child Health Department, Hawassa University, Hawassa, Ethiopia; ^2^ Cardiac Center Ethiopia, Addis Ababa, Ethiopia; ^3^ Internal Medicine Department, Division of Cardiology, Hawassa University, Hawassa, Ethiopia

**Keywords:** ASD, secundum, percutaneous closure, immediate outcome, in-hospital outcome, complications

## Abstract

**Introduction:**

Secundum Atrial septal defect (ASD) is the most common type of ASD. When it is large and hemodynamically significant, it can cause symptoms such as dyspnea, exercise intolerance, and palpitations. Following diagnosis confirmation, an ASD with hemodynamic significance should be closed electively. Percutaneous closure (PC) is an effective treatment option for ostium secundum ASD with adequate rims, despite the potential for several complications. This is the first study in Ethiopia and sub-Saharan Africa to report on percutaneous closure of secundum atrial septal defects (ASD) in children, adolescents, and adults. The study assessed the clinical characteristics, immediate and in-hospital outcomes, and complication rates following PC of secundum ASD.

**Methodology:**

his follow-up study, conducted between October 2023 and January 2024, involved 99 patients who underwent percutaneous closure (PC) of ASD at the Cardiac Center of Ethiopia between January 2013 and January 2023. The patients were divided into two groups based on age: Group 1 included children and adolescents (≤18 years of age; *n* = 42), while Group 2 consisted of adults (>18 years of age; *n* = 57), at the time of device percutaneous closure. The median and interquartile range was used to describe continuous variables. The absolute frequency and percentages were used to describe the categorical variables. The data were shown using tables and graphs. Baseline characteristics of patients of ≤18 years vs. >18 years were compared using the Mann–Whitney *U*-test for continuous variables and the Chi-square or Fisher exact test for categorical data.

**Results:**

There was a female predominance with female to male ratio of 1.3. Compared to Group 1 (children and adolescents of age ≤ 18 years), more patients in Group 2(adults of age > 18 years) experienced symptoms (*p*-value < 0.001). The most common symptoms in adults were easy fatigability and dyspnea (63.2% of adult patients), while the most common symptom in children was recurrent respiratory tract infections (23.8%). Patients in Group 2 had greater pulmonary artery systolic pressure than those in Group 1 (*p*-value < 0.001). Overall, 88.9% of patients attained immediate success; there was no statistically significant difference between the two groups in immediate success rate (*p* = 0.52; Group 1 85.7% vs. Group 2 91.2%). Overall, patients' median length of hospital stay was 2 days (IQR, 2–2.5 days). There was no statistically significant difference between the two groups' median hospital stays [Group 1: 2 (IQR, 2–2.5) and Group 2: 2.5 (IQR 2–2.5); *P*-value = 0.111]. 23.2% of patients experienced complications, with no significant difference between the two groups (Group 1 28.6% and Group 2 19.3%, *p* = 0.28). The most common complications for patients in Groups 1 and 2 were atrioventricular (AV) valve encroachment (4.8) and paroxysmal supraventricular tachycardia (SVT) (5.3%), respectively. The major complication rates in the two groups did not show a statistically significant difference. Group 1 had a rate of 4.8%, whereas Group 2 had a rate of 0% (*p*-value = 0.18). The length of stays significantly increased in both groups in the presence of complications (*p*-value < 0.001).

**Conclusion:**

This study emphasized that Percutaneous Closure of ASD can be successfully performed in resource-limited settings with a high immediate success rate and minimal complications such as AV valve encroachment and paroxysmal SVT. Given that complications impact length of hospital stays, preventing them is crucial.

## Introduction

The interatrial septum, which begins to develop in the fifth week of intrauterine life, effectively divides the left and right atriums (1). Nonetheless, atrial septal defects(ASDs)occur in 1.44–1.66 per 1,000 live births globally; 65–80 percent of these defects are secundum atrial septal defects (2–4). Secundum ASD is mostly asymptomatic, but if it is hemodynamically significant it can cause symptoms including exercise intolerance, dyspnea, and palpitation (5).

ASD closure, whether percutaneous or surgical, is indicated by the following criteria: documented orthodeoxia-platypnoea regardless of shunt size, enlargement of right heart structures irrespective of symptoms, and suspicion of paradoxical embolism in the absence of other causes (4, 6, 7). Following diagnosis confirmation, an ASD with hemodynamic significance should be closed electively. Closure should preferably be done on asymptomatic children between the ages of three and five years (4, 8)

In untreated patients, the risk of right atrial and right ventricular volume overload, heart failure, paradoxical embolization with stroke, atrial fibrillation, right ventricular dysfunction, increased pulmonary vascular resistance, flow-related pulmonary arterial hypertension, increment in size in most patients, and death increases with age (3, 4, 9–11).

Despite significant worldwide disparities in congenital heart disease treatment, many children with ASD survive into adulthood because of advancements in interventions (3, 12, 13). For the majority of patients with secundum ASD, percutaneous closure (PC) is the treatment option, and it produces excellent results (14).

Percutaneous ASD closure results have significantly improved as much as the quality of new devices. Beneficial electrical and geometric cardiac changes can be achieved without the need for cardiopulmonary bypass, scarring and blood transfusions required by surgery. Reduced risk of atrial fibrillation, improved cardiac output and exercise capacity, better functional class, reverse right ventricular remodeling, less tricuspid regurgitation and preservation of both left and right atrial regional myocardial properties are some of the advantages of device ASD closure over surgery (10, 13, 15–18). Furthermore, success rates are higher, ranging from 96 to 98% (3, 19).

However, PC of ASD is associated with various complications, just like any other catheter-based procedure for congenital cardiac diseases (15). Despite being uncommon (<1%), some of the complications require surgery (3, 16, 17). Aortic or atrial perforation or erosion, hemopericardium with tamponade, aortic or mitral valve injury, endocarditis, thrombo-embolism, stroke, and significant residual shunt are among the major complications that can lead to surgery (4, 10, 18).

To the best of our knowledge, there is no existing research on the clinical characteristics of patients, complications, and outcomes of PC of ASD in Ethiopia and sub-Saharan Africa. Therefore, this study is the first to assess these factors in the region. Therefore, this study assessed the clinical characteristics, midterm complication rate, and immediate and in-hospital outcome of patients undergoing PC of ASD at the Cardiac Center of Ethiopia.

## Methods

### Study area and period

This study was conducted at the Cardiac Center of Ethiopia in Addis Ababa, Ethiopia, from October 2023 and January 2024, on patients who had undergone PC of ASD beginning from January 2013 to January 2023. Three interventional cardiologists (two adult and one pediatric) perform ASD device closure.

### Study design

A retrospective, hospital-based follow-up study.

### Eligibility criteria

This study included all secundum ASD patients who underwent percutaneous closure (PC) of ASD at the Cardiac Center of Ethiopia. Those with incomplete charts or medical records were excluded.

### Outcomes of the study

Baseline clinical characteristics, the immediate and in-hospital outcome, complications rate.

### Operative techniques and procedures

Initially, the medical histories, physical exams, electrocardiograms (ECGs), echocardiograms, and chest radiographs (CXRs) of every patient were evaluated. ASD morphology and other important parameters were assessed using standard echocardiographic studies [Transthoracic echocardiography (TTE) in children and TTE and transesophageal echocardiography (TEE) in adults] to determine the feasibility of PC of ASD. Those with secundum atrial septal defect (ASD) presenting a left-to-right shunt, dilation of the right heart chambers, and a rim of 5 mm or greater between the defect and the pulmonary vein, atrioventricular (AV) valves, superior vena cava (SVC), and inferior vena cava (IVC), as well as patients with a small shunt of less than 5 mm who exhibit symptomatic arrhythmia or transient ischemic attacks, including dizziness and syncope, are appropriate candidates for percutaneous closure provided that systolic pulmonary arterial pressure is less than 50% of systolic systemic pressure. Patients with inadequate rims (rim < 5 mm) and severe pulmonary hypertension, with systolic pulmonary arterial pressure greater than 50% of systolic systemic pressure, are excluded from being candidates for the procedure.

After obtaining consent, patients were scheduled for local anesthetic for adults and general anesthesia for children during the trans-catheter closure of ASD. In all cases, right heart catheterization was performed to measure pulmonary pressure. Before the procedure, and after femoral access, all patients received intravenous antibiotics (Ceftriaxone 50 mg/kg single dose) and 50 units/kg/dose of unfractionated heparin via an arm approach. A maximum of 3,000 units of unfractionated heparin was given. After that, angiographic measures were taken. It was preferable to choose a device that was 1–3 mm larger than the defect's diameter. Every device was selected to have a diameter smaller than the septum. In our cases, PC of ASD was accomplished using three different types of devices: the Amplatzer Septal Occluders, the LifeTech CeraFlex TM ASD occluder, and The Cocoon Atrial Septal Occluder.

The device was delivered via a guide wire that passed through the defect. Using the stop-flow technique for balloon sizing, the stretch diameter of the ASD was measured only in the first 8 adult patients who underwent device closure. It was not possible to perform this measurement in all subsequent patients as the balloon stopped functioning thereafter. The appropriate delivery system was used to deploy the ASD device after fluoroscopy and TTE confirmed a satisfactory device position. When the procedure extended longer than sixty minutes, the active clotting time (ACT) was measured and maintained in a constant range of two to three hundred seconds. In situations where the ACT was shorter than 200 s, an additional 50 units/kg/dose of unfractionated heparin injection was given.

Following the procedure, patients underwent a 24-h follow-up in the hospital before being discharged after TTE and ECG evaluations. Initially, a one-month follow-up was scheduled, followed by subsequent appointments every six months for stable patients for the first two years, and then annually thereafter. Transthoracic echocardiography assessed the existence of residual shunt and potential complications. The ECG was used to evaluate arrhythmias. For the first six months following the procedure, the patients were treated with a dosage of 3–5 mg/kg of acetylsalicylic acid. Follow-up was done once a year following the first year. Endocarditis prophylaxis with ampicillin was recommended if the patient undergoes a dental procedure within six months of ASD closure to prevent endocarditis.

### Terms and operational definitions

A residual shunt measuring less than 2 mm was defined as tiny residual. Immediate success was attained if the patient had no residual shunt after PC of ASD. If the patient experienced one or more complications, the label “yes” was applied. If a patient needed immediate surgical or percutaneous intervention after percutaneous closure, it was considered to have a major complication.

### Data collection tool and procedure

A structured questionnaire was used for data collection. The data-collection instrument was developed after a thorough review of a body of literature. Experienced healthcare professionals oversaw the administration and data collection. Supervisors and data collectors were given a two-day training. Objectives of the study, instrument content, data collection technique, ethical considerations, and data collection tasks were given a lot of attention.

### Data quality control

A three-day training was given for data collectors and supervisors to ensure data quality. The focus was on data collection techniques, the objective of the study, and ethical issues. To ensure validity and consistency, the instrument was pretested on 5% of the patients before data collection. The principal investigator-led and supervised the entire data collection process.

### Data processing and analysis

The data was manually examined to ensure its accuracy. The data was coded and then exported to SPSS for Windows and Microsoft Excel 2013, version 25 (SPSS, Chicago, IL, USA) for analysis after being cleaned up with the Epi-data tool, version 4.4.2.1. The 99 consecutive patients who underwent PC starting from January 2013 to January 2022 were categorized into two groups according to age: Group 1 children and adolescents (≤18 years of age; *n* *=* 42), and Group 2 adults (>18 years of age; *n* *=* 57), at the time of PC of ASD. A normality test was conducted using the Shapiro–Wilk test before performing the descriptive statistics for the continuous variables. Categorical variables were described using absolute frequency and percentages. Tables and graphs were used to present the data. Baseline characteristics of patients of ≤18 years vs. >18 years were compared using the Mann–Whitney *U*-test for continuous variables and the Chi-square or Fisher exact test for categorical data. Hospital stays of patients with complications or major complications were compared to those without complications or major complications using the Mann–Whitney *U*-test.

## Results

### Baseline characteristics of patients who underwent percutaneous closure of ASD

**Figure 1:**
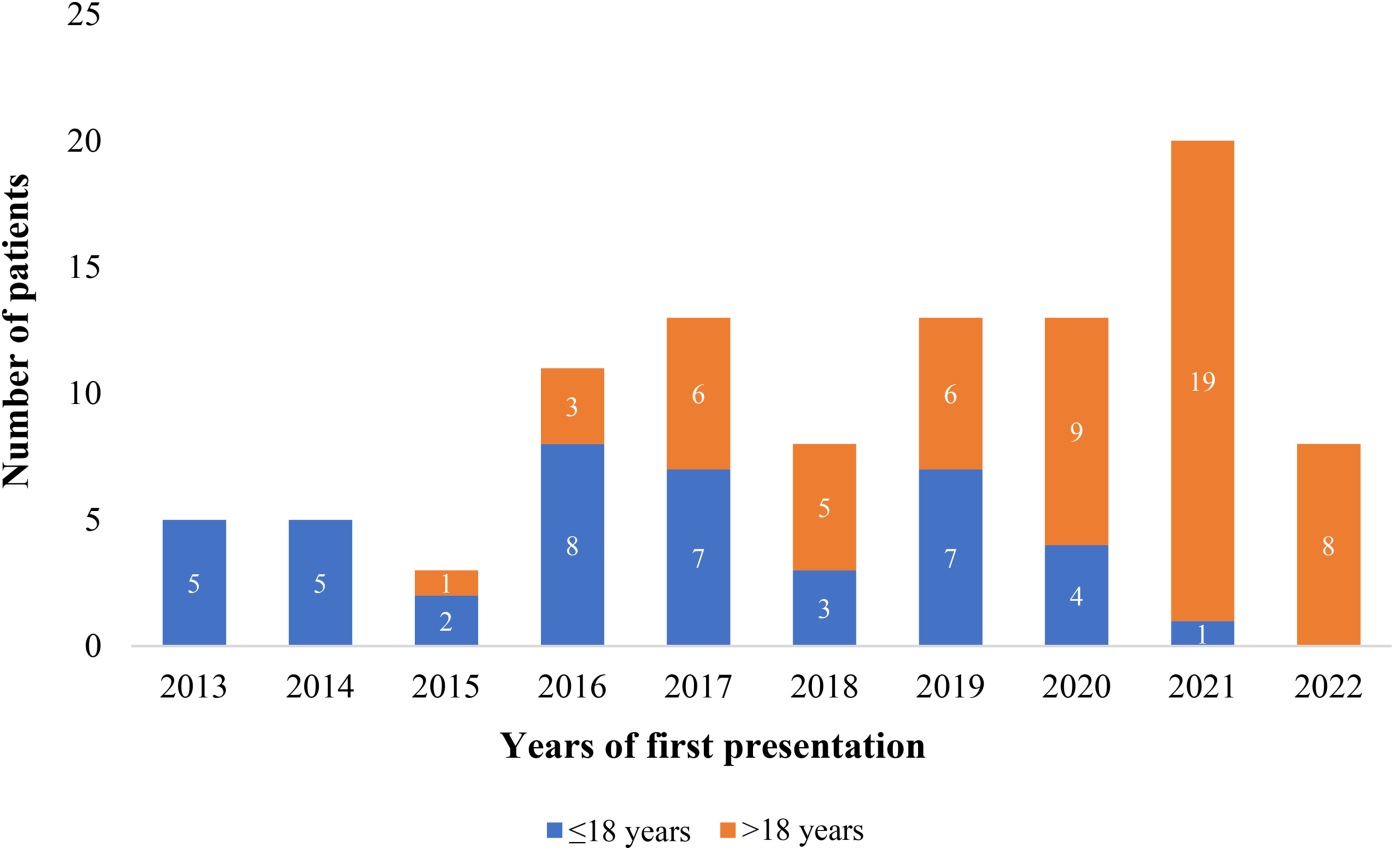
Proportion of patients who underwent PC of ASD at the cardiac center of Ethiopia (January 2013–January 2023).

**Figure 2:**
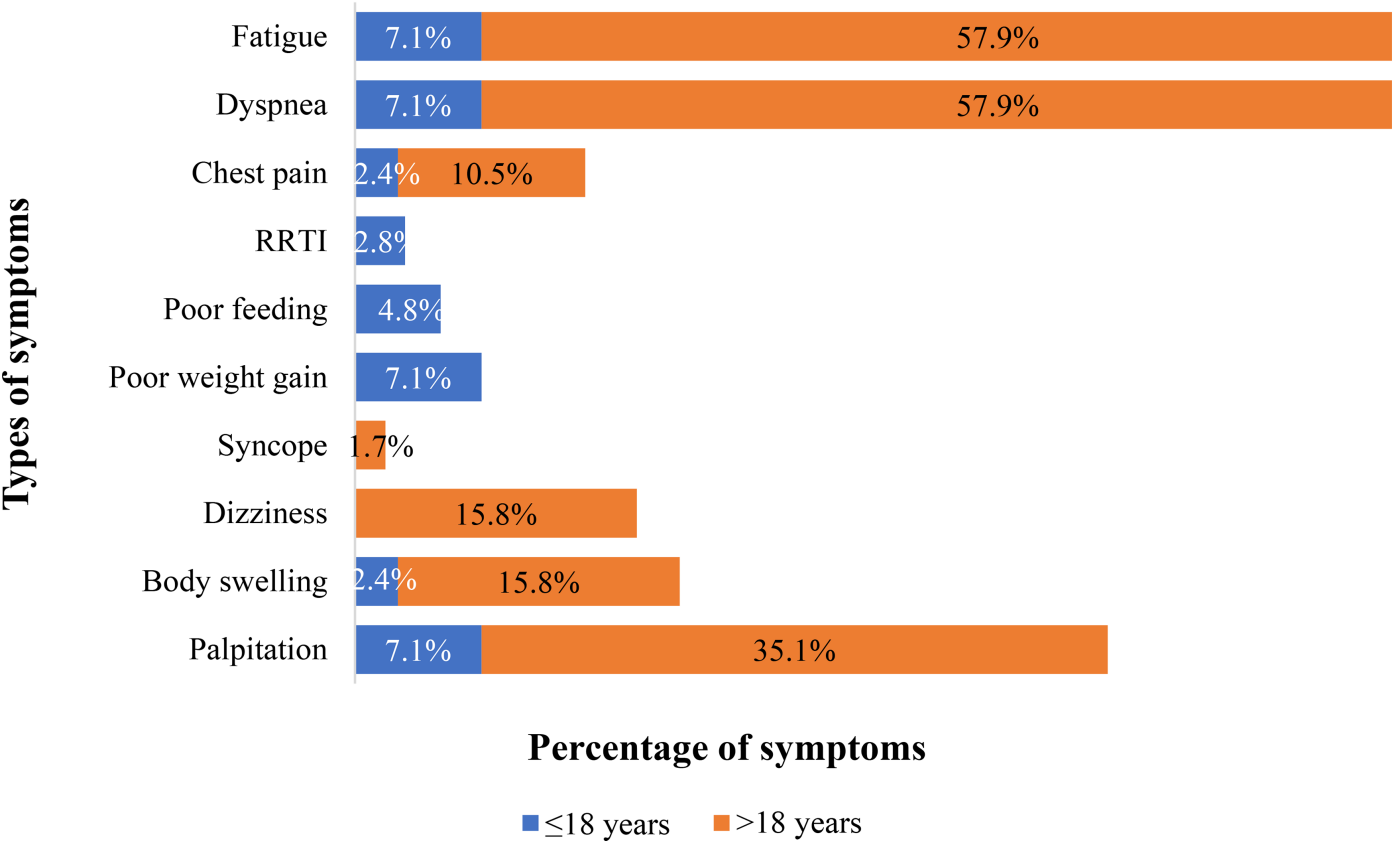
Proportion and type of symptoms at presentation in patients who underwent PC of ASD at the cardiac center of Ethiopia (January 2013–January 2023). 30 patients had one symptom, 13 had two, 19 had three, and 6 had four. *RRTI, Recurrent respiratory tract infections.

Table 1 displays the patients’ sociodemographic characteristics, clinical features, and outcome data. There were 99 patients in total, 42 in Group 1 and 57 in Group 2 (see Figure 1). There is a statistical difference between the gender ratios in the two groups. There was a majority of males in Group 1 and females in Group 2, with *p*-values of 0.006 and female-to-male ratios of 2.2 and 0.68, respectively. The percentage of symptomatic patients in the two groups differs statistically significantly: whereas 90.8% of patients in Group 2 exhibited symptoms, just slightly more than half of patients (50.4%) in Group 1 did (*p*-value < 0.001). For Group 1 patients, the primary complaint was recurrent respiratory tract infection (23.8%), while for Group 2 patients, the primary symptoms were dyspnea (63.2%) and easy fatigability (63.2%) (See Figure 2). Comorbidities were present in slightly more than 1/4 of patients (27.3%) overall. There were seven patients with comorbidities in Group 1. The comorbidities included two patients with global developmental delay, one with Noonan syndrome, one with tiny Patent ductus arteriosus, one with rickets, one with rheumatic valvular heart disease (mild MS), and one with pulmonary tuberculosis. While in Group 2, twenty patients had comorbid conditions: one patient had Deep Vein Thrombosis (DVT) with stroke, one patient had diabetes mellitus, one patient had allergic sinusitis, one patient had asthma, one patient had systemic hypertension, one patient had iron deficiency anemia, one patient had limb deformity, two patients had major depressive disorder, one patient had an epigastric hernia, three patients had moderate PS, one patient had congenital mitral valve prolapse, two patients had morbid obesity, one patient had polycythemia, one patient had rheumatic valvular heart disease (mild MS), two patients had urolithiasis.

**Table 1:** Socio-demographic characteristics, clinical features, and outcome data of patients who underwent PC of ASD at the Cardiac Center of Ethiopia (January 2013–January 2023).

Variable	All	≤18 years	>18 years	*P*-value
Sex, *n* (%)				0.006
Male	43 (43.4)	25 (59.5)	18 (31.5)	
Female	56 (56.6)	17 (40.5)	39 (68.4)	
Age in years, median(IQR)	21 (7–29)	6 (4–10)	26 (21–36)	<0.001
Weight in kg, median(IQR)	43 (20–57)	19 (13.5–26.25)	53 (45.5–60)	<0.001
Height in cm, median(IQR)	156 (115–163)	114 (98–129)	162 (159–167)	<0.001
Comorbidity/ies, *n* (%)				
No	72 (77.8)	35 (83.3)	37 (64.9)	0.067
Yes	27 (27.3)	7 (16.7)	20 (35.1)	
Symptom/s, *n* (%)				<0.001
No	24 (24.2)	19 (45.2)	5 (8.8)	
Yes	75 (75.8)	23 (54.8)	52 (91.2)	
Cardiomegaly in CXR, *n* (%)				0.573
No	28 (28.3)	14 (33.3)	16 (28.1)	
Yes	69 (69.7)	28 (66.7)	41 (71.9)	
Right Axis deviation (ECG), *n* (%)				0.162
No	15 (15.2)	9 (21.4)	6 (10.5)	
Yes	84 (84.8)	33 (33)	51 (89.5)	
Right atrial (RA) size in mm, median(IQR)	40 (29–48)	28.5 (24–35)	45 (40–50)	<0.001
Right Ventricular (RV) size in mm, median(IQR)	34 (25–41)	24 (20–29.25)	40 (34–46)	<0.001
LV ejection Fraction (LVEF) in percent, median(IQR)	65 (60–68)	66 (62–70)	62 (60–65)	0.004
TAPSE in mm, median(IQR)	22 (20–24)	23 (20.75–24.25)	22 (20–24.5)	0.257
Total IAS length in mm, median(IQR)	41.7 (36.7–53.1)	36.4 (31.3–41.7)	50 (41.7–57.1)	<0.001
Deficient Retro aortic rim, *n* (%)				0.756
No	56 (56.6)	23 (54.8)	33 (57.9)	
Yes	43 (43.4)	19 (45.2)	24 (42.1)	
Deficient Posterior rim, *n* (%)				0.571
No	85 (88.9)	35 (83.3)	50 (87.7)	
Yes	14 (14.1)	7 (16.7)	7 (12.3)	
TR severity, *n* (%)				0.02
Mild	27 (27.3)	17 (40.5)	10 (17.3)	
Moderate	60 (60.6)	19 (45.2)	41 (71.9)	
Severe	12 (12.1)	6 (14.3)	6 (10.5)	
Pulmonary systolic pressure in mmHg, median(IQR)	36 (26.25–45.75)	42 (28.3–42.1)	47.5 (40–65)	<0.001
ASD size in mm, median IQR)	18 (14–23)	17 (13–18)	21 (18–26)	<0.001
ASD device type, *n* (%)				0.32
ASO	96 (97)	41 (97.6)	55 (96.5)	
LASO	2 (2)	0 (0)	2 (3.5)	
Cocoon occluder	1 (1)	1 (2.4)	0 (0)	
ASD device size in mm, median(IQR)	18 (15–24)	15 (13–18)	22 (18–27)	<0.001
Device-to-defect ratio, median(IQR)	1.14 (1.1–1.2)	1.15 (1.1–1.21)	1.1 (1.08–1.2)	0.027
Device to total IAS length ratio, median(IQR)	0.49 (0.48–0.5)	0.49 (0.48–0.5)	0.49 (0.48–0.49)	0.353
Fluoroscopic time in minutes, median(IQR)	20 (15–48)	22 (16–42)	20 (14–51.5)	0.542
Procedure time in minutes, median(IQR)	67 (50–90)	67 (55.75–90)	70 (46–87)	0.468
Immediate success, *n* (%)				0.52
No	11 (11.1)	6 (14.3)	5 (8.8)	
Yes	88 (88.9)	36 (85.7)	52 (91.2)	
Follow up duration in years, median(IQR)	3 (1–5)	5 (3.75–7.25)	2 (1–4)	<0.001
Presence of any complications, *n* (%)				0.28
No	76 (76.8)	30 (71.4)	46 (80.7)	
Yes	23 (23.2)	12 (28.6)	11 (19.3)	
Presence of major complications, *n* (%)				0.18
No	97 (98)	40 (95.2)	57(100)	
Yes	2(2)	2(4.8)	0(0)	
Total length of hospital stays in days, median(IQR)	2(2–2.5)	2(2–2.5)	2.5(2–2.5)	0.111

ASD, atrial septal defect; ASO, amplatzer septal occluder; IAS, interatrial septum; LASO, LifeTech CeraFlex™ ASD occluder; TAPSE, tricuspid annular plane systolic excursion; TR, tricuspid regurgitation.

In the majority of our cases, the Amplatzer Septal Occluder was utilized in both groups, with 96 patients (97%) receiving this device. Only one pediatric patient received the Cocoon device, and two adult patients underwent device closure with LifeTech CeraFlex™ ASD occluder (LASO).

As seen in Table 1, the patients in Group 2 had statistically significantly larger values of right atrial size, right ventricular size, pulmonary systolic pressure, interatrial size, ASD device size, and follow-up duration compared to those in Group 1 (*p*-value < 0.001 for each). Group 1 patients had higher median ejection fraction and device-to-defect ratio than Group 2 patients, with the former being 66% vs. 62% (*p*-value = 0.02) and the latter being 1.1 vs. 1.15 (*p*-value = 0.027). The proportion of tricuspid regurgitation severity grade differed statistically between the two groups (*p*-value = 0.04).

**Figure 3:**
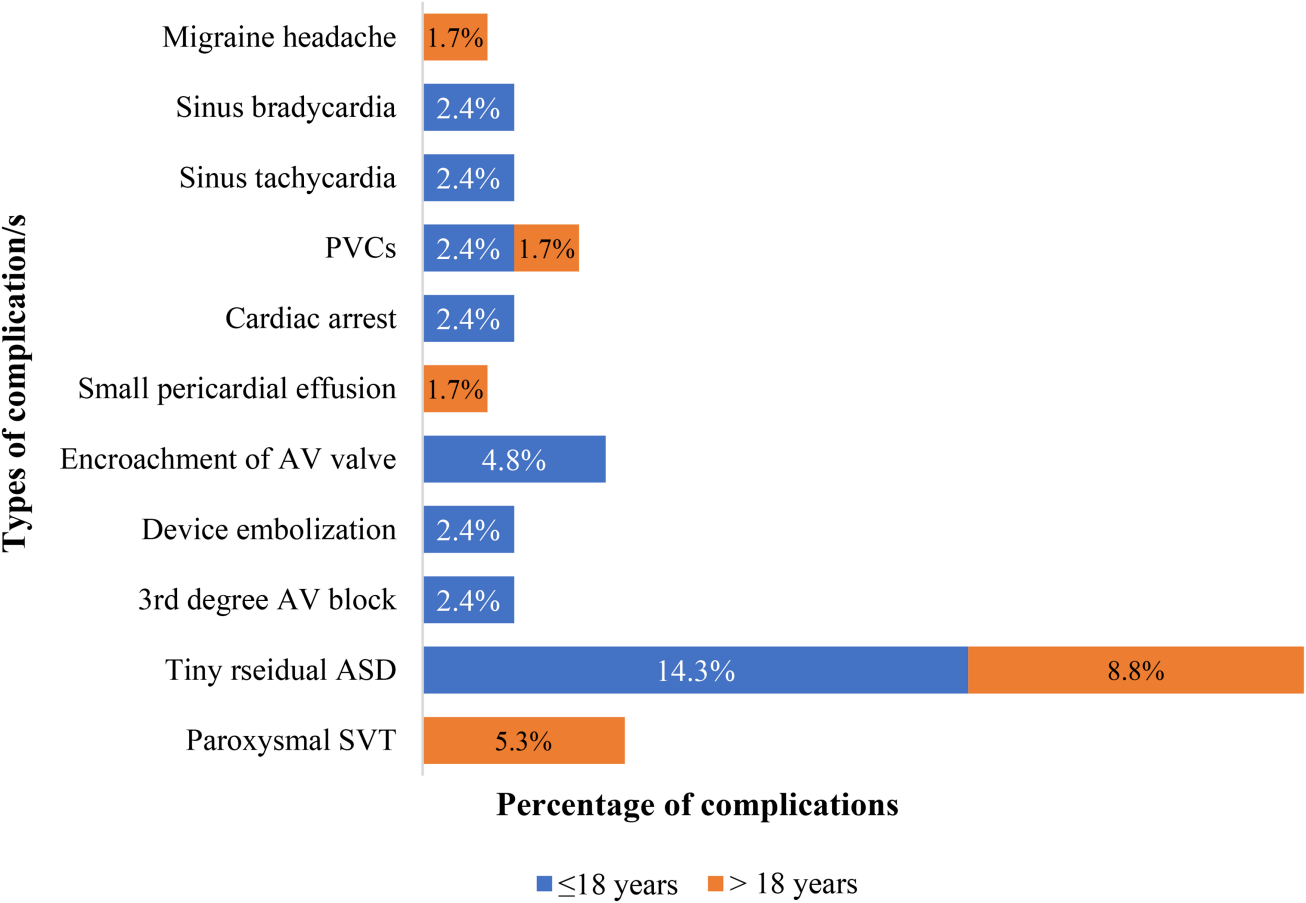
Proportion and type of complications at presentation in patients who underwent PC of ASD at the cardiac center of Ethiopia (January 2013–January 2023). PVCs, premature ventricular contractions; AV, atrioventricular; SVT, supraventricular tachycardia.

Table 1 shows between the two groups, there was no statistically significant difference in the rates of any complications, major complications, and immediate outcomes (*p*-values = 0.28, 0.18, and 0.52, respectively). Figure 3 depicts 25 complications experienced by 99 patients. Paroxysmal SVT was the most common complication in Group 2, affecting three patients, while AV valve encroachment with mild mitral regurgitation was the most common complication in Group 1, affecting two patients. Paroxysmal SVT, PVCs, sinus bradycardia, and sinus tachycardia developed immediately after the procedure and resolved by themselves in both groups in the first 48 h in the hospital. The patient who developed cardiac arrest during the procedure survived after doing CPR. Surgically device retrieval and closure of ASD were performed on a patient who experienced embolization of the device in the left atrium during the procedure. Mild MR that occurred due to mitral valve encroachment didn't show an increment in grade until the end of this study's follow-up period. Spontaneous closure of tiny residual shunts occurred in 98.5% and 99.8% of patients within 1-year and 2-year post-ASD closure time respectively. After four years follow up no patient had residual ASD. A permanent pacemaker was implanted for a patient who developed 3rd-degree AV block immediately after the procedure and the patient with migraine headache (developed 6 months after procedure) has been in follow-up with PO medication (Ibuprofen) intermittently. The small pericardial effusion disappeared after 10 days with no medication. No mortality was documented in either group during the immediate post-procedure or the follow-up period. As presented in Table 2, length of hospital stays were statistically significantly longer in both groups when patients had complications.

**Table 2:** Difference in length of hospital stays between patients with complication/s versus those with no complications in patients who underwent PC of ASD at the cardiac center of Ethiopia (January 2013–January 2023).

	≤18 years		*P*-value
	No complication	With complication/s	
Total length of Hospital stays in days, median(IQR)	2 (2–2.1)	3 (2.5–3.5)	<0.001
	>18 years		
	No major complication	With major complication/s	
Total length of Hospital stays in days, median(IQR)	2 (2–2.5)	3 (2.5–3.5)	<0.001

## Discussion

This study assessed the clinical characteristics and immediate and midterm outcomes of patients who underwent percutaneous ASD closure at the Cardiac Center of Ethiopia. It has revealed a numerically a lower immediate success rate than comparable studies conducted in different settings, but the midterm outcome showed comparable clinical characteristics and a rate of complications.

Similar to our study, but with a different proportion, research from the USA, Turkey, Switzerland, and Korea all demonstrate a female predominance (19–23). There are several hypotheses as to why ASD is more common in females, including the role of sex hormones in the development of the cardiovascular system, sex differences in response to stresses (both endogenous and exogenous), and differences in x and y linked genes in the development of the cardiovascular system (24).

The majority of adult patients were symptomatic, with dyspnea being the most common symptom (66%) according to a study conducted in Canada, which supports our study's finding that symptoms mostly occur during adulthood (25). Age-related increases in the risk of atria arrhythmias, pulmonary hypertension, and a decrease in left ventricular compliance accelerate the development of symptoms in older adults.

Ten Percent of our patients (all Group 1 patients) experienced recurrent respiratory tract infections, a finding also reported in studies conducted in pediatric patients in China and Italy, where patients experienced these infections in 23.9% and 16.7% of cases, respectively (26, 27). Recurrent lower respiratory tract infections are the ultimate consequences of pulmonary over-circulation brought on by large ASD.

The majority of patients in our study—69.7% of children and 66.7% of adults—had cardiomegaly. This finding is corroborated by multicenter studies conducted in the USA (97%), Canada (91%), and Iran (61.3%) (25, 28, 29). Our right axis deviation percentage of 84.8% was significantly higher than the study conducted in the USA, which found a percentage of 28.7% (30). Variation in the proportion of individuals with cardiomegaly and right axis deviation is determined by differences in the number of patients with hemodynamically significant ASD among studies.

In line with our research, a comparative study conducted in Turkey revealed that children have a higher device-to-defect ratio (31).

A device's embolization, erosion, and encroachment onto surrounding cardiac tissues are among the complications that deficient rims may pose (32).

A deficient retro aortic rim was present in 43% of our patients, that was comparable to 40% report in a multicenter study conducted in the USA, but it is significantly higher than 11.5% in another USA and 16.8% in Iran, lower than 75% in France, 54.6% in the UK, and 63% in the USA (28, 33–37). Our study's 14% deficient posterior rim percentage was lower than 25% from France but significantly higher than 0.5% from the UK and 1.1% from China (26, 34, 35).

ASD device closure has an immediate success rate that varies from 79 to 100%; the majority of them had an immediate success rate greater than 90% (20, 26–28, 34, 38–41). Compared to most studies, our initial 88.9% success rate was lower than most studies from other centers. The immediate success of ASD device closure is dependent on a precise ASD morphological assessment and a suitable device selection (42).

Numerous studies reported a 0–26.7% complication rate with no mortality (21, 26, 27, 30, 39, 43–52). The reported major complications rate is 0–1.5 percent, while minor complications rate of 0–11.2% (26, 27, 30, 34, 44, 47, 48, 50, 53–57). Both complication and mortality rates in our study are within this range. The type of device, age, morphology of the defect, and experience of the interventional cardiologists all influence the variation in the complication rate among studies.

Similar to this study, device embolization into the heart's chambers is the most common major complication across studies, according to a meta-analysis conducted to assess the complications of ASD device closure (57). Device embolization was the most frequent major complication in the USA (0.2%), Belgium (1%), Germany (0.3%), another German study (1.6%), Thailand (2.6%), Turkey (0.48%), Italy (20.6%), Finland (0.1%), India (0.54%), other India (9%), Chinese (1.1%) studies (21, 26, 28, 48, 53, 56, 58–62). Similar to our research, a comparative study conducted in Canada revealed no association between the age group and the embolization rate (63).

Complete AV Block requiring permanent pacemaker implant was the second major complication in our study that was rarely reported elsewhere. USA single and multicenter studies reported only one case of Complete AVB requiring permanent pacemaker implant (30, 53).

Transient arrhythmias were observed in 7.1% of patient, namely sinus bradycardia, sinus tachycardia, SVT, and PVCs, and is comparable to the 0%–9% rate reported in the literature (26–28, 30, 34–36, 43, 46, 51, 55, 59, 64–67). Additionally, there are only a few reports of cardiac arrest after ASD device closure comparable to our study (53).

Device encroachment on AV valves caused mild MR in 2 of our cases, which is consistent with limited reports of mild-to-moderate MR in children and adults both in Poland (1.34%) and India (0.54%) (53, 68).

The pericardial effusion rate in our study falls between 0.1% and 5.8%, a range that was found in studies from both developed and developing nations (28, 29, 35, 48, 52, 56, 61, 62, 67, 69).

Although the cause of migraine headaches following the closure of ASD devices is unknown, there have been infrequent reports from a multicenter study conducted in the USA (1.9%) and a single UK study (3.9%), similar to our study (of which we reported 1%) (28, 35).

Residual shunt rates after ASD percutaneous closure show ample variation among reports from Argentina (8.8%), 13% in Italy, 4.3% in Turkey, 5% in Brazil, and 11.1% in our own study. However, all of them, including ours, reported spontaneous closure between 24 h and 4 years after ASD percutaneous closure (20, 27, 38, 51).

Consistent with our findings, a Swedish study revealed that the occurrence of immediate complications following the closure of an ASD increases hospital stay duration (44). The more straightforward explanation for this would be because additional care is required to treat complications, besides what's customary needed as immediate routine for percutaneous closure procedures.

## Conclusion

This study emphasizes the effective performance of Percutaneous ASD Closure in resource-limited environments for both children and adults. The procedure has shown a high immediate success rate and minor complication rates such as minimal risk of encroachment on the AV valve and paroxysmal SVT. Major complications occurred in only two pediatric patients, namely device embolization and third degree AV block. As complications can lead to prolonged hospital stays, it is crucial to focus on their prevention.

## Data Availability

The raw data supporting the conclusions of this article will be made available by the authors, without undue reservation.
